# Whole Genomic Analysis and Comparison of Two Canine Papillomavirus Type 9 Strains in Malignant and Benign Skin Lesions

**DOI:** 10.3390/v12070736

**Published:** 2020-07-08

**Authors:** Chia-Yu Chang, Nanako Yamashita-Kawanishi, Sonoka Tomizawa, I-Li Liu, Wei-Tao Chen, Yen-Chen Chang, Wei-Hsiang Huang, Pei-Shiue Tsai, Kinji Shirota, James K Chambers, Kazuyuki Uchida, Takeshi Haga, Hui-Wen Chang

**Affiliations:** 1 School of Veterinary Medicine, National Taiwan University, Taipei 10617, Taiwan; flywinds11@gmail.com (C.-Y.C.); liuili@ntu.edu.tw (I.-L.L.); b04609022@ntu.edu.tw (W.-T.C.); yenchenchang@ntu.edu.tw (Y.-C.C.); whhuang@ntu.edu.tw (W.-H.H.); psjasontsai@ntu.edu.tw (P.-S.T.); 2 Graduate Institute of Molecular and Comparative Pathobiology, School of Veterinary Medicine, National Taiwan University, Taipei 10617, Taiwan; 3 Division of Infection Control and Disease Prevention, Graduate School of Agricultural and Life Sciences, The University of Tokyo, Tokyo 113-0033, Japan; nanakoyamashita1906@g.ecc.u-tokyo.ac.jp (N.Y.-K.); sonoka.colorfulsky@gmail.com (S.T.); 4 Institute of Veterinary Clinical Science, School of Veterinary Medicine, National Taiwan University, Taipei 10617, Taiwan; 5 Laboratory of Veterinary Pathology, School of Veterinary Medicine, Azabu University, Kanagawa 252-5201, Japan; shirota@azabu-u.ac.jp; 6 Department of Veterinary Pathology, Graduate School of Agricultural and Life Sciences, The University of Tokyo, Tokyo 113-0033, Japan; achamber@mail.ecc.u-tokyo.ac.jp (J.K.C.); auchidak@mail.ecc.u-tokyo.ac.jp (K.U.)

**Keywords:** canine papillomavirus type 9 (CPV9), E6, E7, oncoprotein, squamous cell carcinoma (SCC), E2

## Abstract

Papillomaviruses (PVs) usually cause benign proliferative lesions in the stratified epithelium of various animal species. However, some high-risk types of PVs have been proven to lead to malignant transformations. In dogs, several canine papillomaviruses (CPVs) have been identified in malignant lesions and are suggested as one of the risk factors for the development of squamous cell carcinomas (SCCs). In the present study, the full genomes of two CPV9 strains from recurrent SCCs of Dog 1 and skin viral papilloma (viral plaque) of Dog 2 were sequenced. Alignment of the two CPV9 sequences with the genome of the reference CPV9 strain (accession no. JF800656.1) derived from a solitary pigmented plaque was performed. Compared with the reference strain, a 27 bp in-frame insertion in the E1 gene was identified in both CPV9 strains in this study. In comparison with the CPV9 strains derived from benign lesions, the CPV9 from the SCCs of Dog 1 exhibited a 328 bp deletion at the 3′ end of the E2 and spacer sequence, which encoded a truncated deduced E2 protein and a chimeric E8^E2 protein. However, there was no difference in the mRNA expression levels of viral oncoproteins of E6 and E7 between the two CPV9 cases, suggesting that the oncogenesis of CPV9 for malignant transformation might be different from that of human papillomaviruses. The roles of E2 and E8^E2 deleted CPV9 in the oncogenesis of benign and malignant lesions should be further investigated.

## 1. Introduction

Papillomaviruses (PVs) were first identified from cutaneous lesions and linked to human cancer in the nineteenth century [1,2]. They are small double-strained DNA viruses that can infect various animal species and have strong species and tissue tropism [3]. Through direct or indirect contacts, the PVs are able to reach basal cells of the epithelium through open wounds and massively propagate when the cells undergo squamous differentiation [4,5]. Papillomavirus infections are usually asymptomatic or cause proliferative cutaneous lesions such as papillomas, pigmented plaques, and warts [3,5]. Generally, these benign hyperplastic lesions can be spontaneously eradicated by elevated host innate immunity as well as cell-mediated immunity [6,7,8]. However, some specific types of PVs, such as human papillomavirus (HPV) types 16 and 18, have been proven to be ‘high-risk’ oncogenic viruses that cause malignant cutaneous and genital neoplasms in humans [9].

The complete genome of PVs is approximately 7.9 kb, encoding two late proteins (L1 and L2), five to seven early proteins (E1, E2, E4, E5, E6, E7, E8), and a long control region (LCR) [10]. L1 is the target gene for phylogenetic analysis and encodes the major capsid protein of PVs [10,11]. The L1 protein (major capsid protein) and L2 protein (minor capsid protein) are packaged into icosahedral virions of approximately 50 nm, and thus are a common target for vaccine development [11,12]. Based on knowledge about the HPVs, the early proteins are multi-functional regulatory proteins that generally participate in the different stages of viral replication, transcription, and oncogenesis [4]. The E1 protein acts as a helicase and can be recruited by the E2 protein [13]. The E2 protein is an essential regulatory protein for PVs and participates in controlling transcription, viral genome replication, cellular apoptosis, and cellular gene expression [14,15,16]. Serving as a DNA-binding protein, E2 uses its C-terminus to dimerize and binds to a specific motif in the long control region (LCR) with the E1 protein, a viral DNA helicase [17,18,19]. Moreover, E2 can either activate or repress viral replication and transcription depending on whether it has high or low expression levels [20]. An alternative short reading frame of E1, which is named E8, can form the E8^E2 transcript, becoming a potent repressor of viral transcription and replication [14,16,21]. The E4 protein may participate in the breakdown of cellular cytokeratin networks and facilitate the release of the virus [22]. The E6 and E7 proteins are well-known oncoproteins that mainly serve as inhibitors of the cellular tumor suppressor protein p53 and repressors of the retinoblastoma (Rb) tumor suppressor family, respectively [23,24].

In veterinary medicine, more than 112 types of non-human PVs have been reported in 54 different animal species, including companion animals, livestock, and wild animals [25]. Among these PVs, bovine papillomavirus (BPV) types 1, 2, 4, and 13, equus caballus papillomavirus (EcPV) type 2, and feline papillomavirus (FcaPV) types 2 and 3 have also been reported to be causative agents of malignant neoplasms such as squamous cell carcinoma (SCC), Bowenoid in situ carcinoma (BISC), and transitional cell carcinoma [3,26,27]. In dogs, 20 types of canine papillomavirus (CPV) have been identified and categorized into three different genera: Lambda-, Tau-, and Chi-papillomavirus [5,28,29]. CPV1, also known as canine oral papillomavirus (COPV), and CPV13 usually cause benign hyperplastic lesions in the oral cavity of dogs [3]. The remaining types of CPVs ordinarily cause benign proliferation on the skin of dogs. However, CPV1, together with CPV2, CPV3, CPV7, CPV9, CPV12, CPV15, CPV16, and CPV17 are also depicted in lesions with malignant transformations [3,30,31,32].

In the present study, the full-length genomes of a pair of CPV9 strains from Dog 1, which had been included in our previous study, with recurrent SCCs [32] and Dog 2 with viral plaque were sequenced to investigate the role of viral genes in the dramatically different pathological phenotypes of the two CPV9 strains. The messenger RNA (mRNA) expression levels of the oncoproteins E6 and E7 were also evaluated and compared between these two CPV9 strains to study the potential mechanism of viral oncogenesis in CPV9.

## 2. Materials and Methods

### 2.1. Sample Collection and Ethical Statement

- Dog 1: Fresh collected and formalin-fixed paraffin-embedded (FFPE) tissue samples from Dog 1 with recurrent SCCs were obtained from the animal hospitals at National Taiwan University in 2018. The sample collections were performed following the guidelines of and under the permissions of the Institutional Animal Care and Use Committees (IACUCs) of National Taiwan University (NTU) with the permission numbers NTU107-EL-00165.
- Dog 2: The FFPE sample from Dog 2 was obtained from the Department of Veterinary Pathology, The University of Tokyo (UT) in 2018 which was deposited for diagnostic purposes and to enable adequate disease prevention strategies. All surgical procedures were performed by educated veterinarians in accordance with Japanese law.

Both dog owners were informed and agreed to the aims and details of the present study.

### 2.2. Case Information

The first case (Dog 1), who had been included in our previous study [32], was an 11-year-old neutered male Schnauzer with multiple skin masses on the flank and inguinal region. The masses were highly pigmented with a hairless cutaneous surface, two of which were discovered on the digits of the forelimb and hindlimb, three masses were located on the bilateral inguinal region, and the rest (more than five masses) were on the trunk. The animal had surgical removal of masses more than eight times in four years, but the masses recurrently grew on the body. The second case (Dog 2) was a 9-year-old neutered male Pomeranian with a single abdominal pigmented viral plaque, no recurrence was observed.

### 2.3. Histological Descriptions and Immunohistochemistry Staining (IHC)

The histopathological diagnoses were determined by veterinary pathologists at the Graduate Institute of Molecular and Comparative Pathobiology at NTU and the Laboratory of Veterinary Pathology at UT. IHC staining was performed as previously described [32]. Briefly, the slides were de-paraffinized in non-xylene (Muto Chemical, Tokyo, Japan) and re-hydraded in serially diluted ethanol. Antigen retrieval was performed in a Triology solution (Cell Marque, Rocklin, CA, USA) at 95 °C for 10 min. Then, the slides were blocked with 10% normal goat serum for 1 h and probed by 1-hour incubation with 100× diluted mouse anti-HPV antibodies (BPV-1/1H8 + CAMVIR; catalog no. ab2417; Abcam, Cambridge, UK). After blocking the endogenous peroxidase activity with 3% H_2_O_2_ and repeated washing, the slides were incubated with goat anti-mouse antibody conjugated with horseradish peroxidase (HRP; Dako, Santa Clara, CA, USA) and incubated for 1 h. The DAB detection buffer (Dako) was used to obtain signals, and hematoxylin was also used to depict the morphology of the tissues.

### 2.4. DNA Extraction from FFPE and Fresh Tissue

To extract the DNA from fresh and FFPE tissues, 250 mg of fresh tissue from Dog 1 and serial sections (in total 40 µm) of the FFPE blocks from Dog 2 were obtained. For the FFPE tissues, the de-paraffinization process was performed by repeated washes with non-xylene (Muto Chemical) and 99% ethanol (Sigma–Aldrich, St. Louis, MO, USA) as previously described [32]. The DNeasy Blood and Tissue Kit (Qiagen, Hilden, Germany) was used. Following the manufacturers’ protocols, both the fresh and FFPE tissues were digested with proteinase K (Qiagen, Hilden, Germany) in ATL buffer (Qiagen) at 56 °C for 14–16 h. The samples from FFPE tissue were treated for another 1 h at 90 °C to eradicate modifications on the DNA from the formaldehyde. Then, both samples were mixed with AL buffer (Qiagen) and 99% ethanol (Sigma-Aldrich). The supernatant was transferred into the DNA-binding column and briefly centrifuged to empty the column. Following the washing procedures with AW1 buffer (Qiagen) and AW2 buffer (Qiagen) and a brief drying centrifuge, the DNA from both samples was eluted with 50 µL of PCR-grade water. The DNA samples were stored at −20 °C until use.

### 2.5. Polymerase Chain Reaction (PCR) for the Detection and Full Genome Sequencing of CPV

To detect the genes of CPVs in the extracted DNA samples, the degenerative primer pair CP4/CP5 (forward: 5′ -ATGGTACARTGGGCATWTGA-3′; reverse: 5′-GAGGYTGCAACCAAAAMTGRCT-3′), targeting the relatively conserved area of PVs, was used [33]. For the full genome sequencing of CPV9, different primer pairs were designed based on the published reference strain of CPV9 (Accession no. JF800656.1). The primer pairs used in the full genome sequencing of CPV9 from the fresh tissue of Dog 1 are listed in Supplementary Table S1, while the primer pairs used for sequencing the genome of CPV9 from FFPE tissue of Dog 2 are listed in Supplementary Table S2. PCR was performed under the same conditions as previously published, but with some modifications [32]. Briefly, 5 µL of 10-fold PCR buffer (Invitrogen, Thermo Fisher Scientific, Waltham, MA, USA), 1 µL of 10 mM dNTP mixture (Invitrogen), 1.5 µL of 50 mM MgCl (Invitrogen), 1 µL of 10 mM forward primer, 1 µL of 10 mM reverse primer, 0.5 µL of Taq DNA polymerase (Invitrogen), 37.5 µL of PCR-grade water, and 2.5 µL of template DNA were mixed for each reaction. The PCR steps were as follows: 94 °C for 3 min; 35 cycles of 94 °C for 45 s, 55 °C for 30 s, and 72 °C for 1–2 min (depending on the length of the amplicons); and a 5 min final extension at 72 °C. The expected size of the amplicon using the CP4/CP5 primer was approximately 450 base pairs (bp). The expected sizes of the amplicons using different specific primers are also listed in Supplementary Tables S1 and S2.

### 2.6. Sequence Analysis

The full genome sequences of each CPV9 strain were analyzed and assembled using Molecular Evolutionary Genetics Analysis Version 7.0. Multiple gene alignment was also achieved using the online software on the NCBI BLAST website and the DNASTAR software (Lasergene, Madison, WI, USA).

### 2.7. RNA Extraction and Genomic DNA Eradication from FFPE Tissue

To evaluate the viral messenger RNA expressed in the tissue, the RNA from both samples was extracted from FFPE tissue using the RNeasy FFPE Kit (Qiagen). Forty micrometer thick tissue samples from the FFPE blocks were sectioned. Following the de-paraffinization procedure of washing the tissue with non-xylene (Muto Chemical) and 99% ethanol (Sigma -Aldrich), the tissues were lysed in 240 µL PKD buffer (Qiagen) with 10 µL proteinase K (Qiagen) at 56 °C for 15 min and then at 80 °C for another 15 min. After brief centrifugation, the supernatant was incubated with 10 µL DNase I (Qiagen) in DNase Booster Buffer at room temperature for 15 min to preliminarily remove the genomic DNA. After mixing with 500 µL RBC buffer (Qiagen) and 1.2 mL 99% ethanol (Sigma -Aldrich), the supernatant was added to an RNeasy MinElute spin column (Qiagen) and washed twice with RPE buffer (Qiagen). The RNA from two FFPE samples was eluted in 30 µL RNase-free water. To completely remove viral DNA from the RNA sample, further DNA eradication was achieved using RNAse-Free DNase Set (Qiagen). Following the manufacturer’ s protocol, 2.5 µL DNase I (Qiagen) was added to 10 µL RDD buffer into the extracted RNA and incubated at room temperature for 10 min. Then, the RNA samples were cleaned up by mixing the RNA with 350 µL RLT buffer and 250 µL of 99% ethanol and applied to an RNeasy Mini spin column (Qiagen). After washing the columns with RPE buffer, the pure RNA samples were eluted in 20 μL RNase-free water. The RNA samples were stored at −80 °C until use.

### 2.8. Primer-Specific cDNA Synthesis of E6 and E7

Primer-specific reverse transcription was performed using the Transcriptor First Strand cDNA Synthesis Kit (Roche, Basel, Switzerland). The reverse primers for partial E6 and E7 reverse transcripts were designed and utilized: CPV9E6mRNA-R: 5′-CCAGGCAGTCTGTACACCTC-3′; and CPV9E7mRNA-R: 5′-CAATCCTGATTCCACGACCG-3′. The internal control for dog ribosomal protein S5 (RPS5) was also included: RPS5-R: 5′-CCTGATTCACACGGCGTAG-3′ [34].

For each reaction, 1 µg total RNA, 1 µL 10mM reverse primer, and a variable volume of RNase-free water were prepared for a total reaction volume of 13 µL. Then, the mixtures were heated at 65 °C for 10 min to denature the secondary structure of the RNA and put on ice immediately after heating. Five microliters of Transcriptor Reverse Transcriptase Reaction Buffer (Roche), 0.5 µL Protector RNase Inhibitor (Roche), 2 µL 10 mM deoxynucleotide mix (Roche), and 0.5 µL Transcriptor Reverse Transcriptase (Roche) were directly added into each mixture. Following a short incubation at 55 °C for 30 min and 85 °C for 5 min, the primer-specific cDNA fragments were synthesized.

### 2.9. The Detection of mRNA Expression Level of E6 and E7 by PCR and qPCR

Viral mRNA expression was evaluated by PCR and qPCR. The primers designed for detecting E6 were CPV9E6mRNA-F: 5′-GCGTGTGCAAGGAATATCTGC-3′ and CPV9E6mRNA-R: 5′-CCAGGCAGTCTGTACACCTC-3′; for E7 they were CPV9E7mRNA-F: 5′-ACCACTTGACAACCTCTGGTG-3′ and CPV9E7mRNA-R: 5′-CAATCCTGATTCCACGACCG-3′; for the internal control of mRNA, primers targeting ribosomal protein S5 (RPS5) were used: RPS5-F 5′-TCACTGGTGARACCCCCT-3′ and 5′-CCTGATTCACACGGCGTAG-3′ [34].

The reverse primers used in PCR were the same primers used for primer-specific reverse transcription. The PCR reaction was conducted with amaR OnePCR (GeneDireX, Taichung, Taiwan). For each reaction,10 µL amaR OnePCR Buffer (GeneDireX), 1 µL 10 mM forward primer, 1 μL 10 mM reverse primer, 2 µL cDNA template, and PCR-grade water for a total of 20 μL were mixed. The process in the thermal cycler was 94 °C for 3 min; 35 cycles of 94 °C for 30 s, 50 °C for 30 s, and 72 °C for 30 s; and a 5 min final extension at 72 °C. Except for the blank negative controls for the PCR reaction, a viral DNA sample was used as a positive control. Furthermore, to confirm that no viral DNA contaminated the mRNA, the RNA samples (before reverse transcription) from Dog 1 and Dog 2 were also included as negative controls. The predicted sizes of the amplicons for E6, E7, and RPS5 were 130 bp, 137 bp, and 141 bp, respectively. After electrophoresis in agarose gel, the amplicons were sent for sequencing. For qPCR, the TB Green® Advantage® qPCR Premix (TakaraBio, Shiga, Japan) was used. For each reaction, 10 µL TB Green Premix (TakaraBio), 0.5 µL 10 mM forward primer, 0.5 µL 10 mM reverse primer, 2 µL cDNA template, and PCR-grade water for a total of 20 µL were mixed. The process in the thermal cycler was 40 cycles of 95 °C for 5 s and 60 °C for 30 s. The signals were detected using a CFX96 Touch Realtime System (Bio-Rad, California, U.S.A.). The E6, E7, and RPS5 qPCR reactions with melting temperature of 82.0 °C, 83.5 °C, and 85.5 °C were defined as positive, respectively.

## 3. Results

### 3.1. Histological Descriptions and Immunohistochemistry Staining

**Figure 1:**
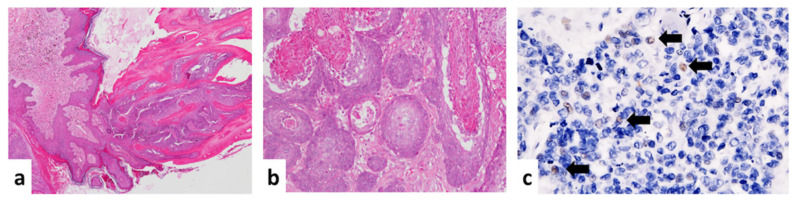
Histological images of papillomas and squamous cell carcinomas (SCCs) of Dog 1 under hematoxylin and eosin staining (H&E) and immunohistochemistry staining (IHC). (**a**) Multiple masses taken from the trunk and digits were diagnosed as papillomas using H&E (40 ×). (**b**) The masses taken from the inguinal region were diagnosed as squamous cell carcinomas (SCCs) with a stromal invasive pattern, moderate keratinization, and nuclear pleomorphism under H&E (100 ×). (**c**) Antigen-specific IHC was performed using anti-HPV antibodies (BPV-1/1H8 + CAMVIR; catalog no. ab2417; Abcam, Cambridge, UK). Scattered positive signals were observed (400 ×).

**Figure 2:**
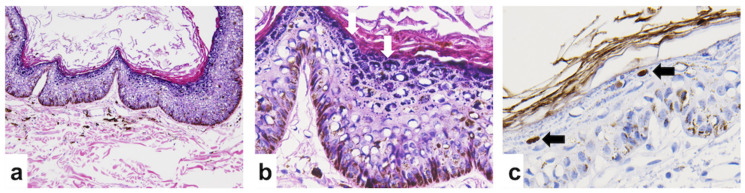
Histological images of a viral papilloma of Dog 2 under hematoxylin and eosin (H&E) staining and immunohistochemistry (IHC) staining. The benign viral papilloma was taken from a cutaneous mass growing on the abdomen. (**a**) Hyperpigmented and proliferative epithelia were observed under H&E (40 ×). (**b**) Several eosinophilic intra-nuclear inclusion bodies (labeled by arrows) were identified in the stratum granulosum (200 ×). (**c**) Antigen-specific IHC was performed using anti-HPV antibodies (BPV-1/1H8 + CAMVIR; catalog no. ab2417; Abcam, Cambridge, UK). Positive signals (labeled by arrows) were observed on the viral inclusions (400 ×).

In Dog 1, multiple skin masses on the trunk and digits were diagnosed as cutaneous horns and multicentric papillomas covered by hyperpigmented squamous epithelium (Figure 1a). The inguinal masses from Dog 1 were diagnosed as squamous cell carcinomas (SCC) with the features of moderate keratinization, an obvious deep invasive pattern, moderate nuclear pleomorphism, and a low mitotic rate; thus, it was categorized as grade 2 SCC based on the Invasive Front Grading System (Figure 1b) [35]. Local lymphatic invasion was also observed in the inguinal masses from the last surgical specimens. No inclusion bodies were found in Dog 1. The IHC staining using the PV-specific antibody depicted scattered brownish positive signals (Figure 1c). According to the feature of the PVs, only the intranuclear signals were interpreted as positive. In Dog 2, the abdominal mass was diagnosed as a viral papilloma lined with hyperpigmented epithelium with the appearance of obvious eosinophilic intra-nuclear inclusion bodies in the stratum granulosum (Figure 2a,b). Intranuclear positive signals were detected by IHC staining in Dog 2 (Figure 2c).

### 3.2. Complete Nucleotide Sequence of CPV9

The viral genomic DNA was sequenced, assembled, and submitted to the National Center for Biotechnology Information (NCBI) with the GenBank accession numbers MT265225 and MT265226, respectively. The full genomic sequence of CPV9 from Dog 1 and Dog 2 consisted of 7572 base pairs (bp) and 7900 bp, respectively, which were different from the complete genomic length of the reference CPV9 strain (Accession no. JF800656.1; 7873 bp). The nucleotide alignment of the complete sequences among these three CPV9 strains indicated that while the sequence identity between CPV9 strains from Dog 1 and Dog 2 was 95.4%, the sequence identities of CPV9 strains from Dog 1 and Dog 2 were 95.3% and 99.4%, respectively, as compared to the reference CPV9 strain.

### 3.3. Open Reading Frames (ORFs) and Deduced Amino Acid Comparisons

**Figure 3:**
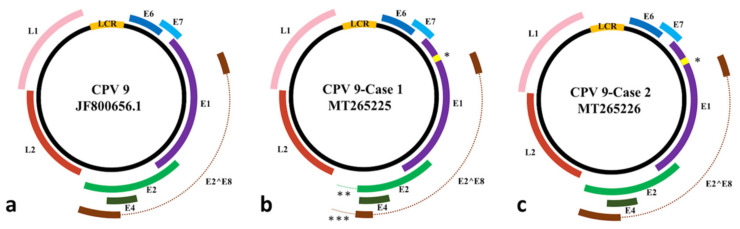
The reprehensive images of viral genome with coding regions of canine papilloma virus type 9 (CPV9). (**a**) The viral genome of reference CPV9 (JF800656.1) strain. (**b**) The viral genome of E2-deleted CPV9 (MT265225) strain from Dog 1, which was diagnosed as squamous cell carcinoma (SCC). (**c**) The viral genome of CPV9 (MT265226) strain from Dog 2, which was diagnosed as benign viral papilloma. LCR: long control region; *: the 27 bp in-frame insertion on the E1 gene; **: the 328 bp deletion covering partial E2 gene; ***: the 328 bp deletion covering partial E2^E8 gene.

Similar to the published canine papillomaviruses, the CPV9 strains identified in Dog 1 and Dog 2 had seven ORFs encoding two late proteins, L1 and L2, and six early proteins, E1, E2, E4, E6, E7, and E2^E8. Schematic diagrams of each predicted ORF of each CPV9 strain are presented in Figure 3. The positions of each ORF are listed in Table 1, and the point mutations, insertions, and deletions of the DNA sequences and their corresponding deduced amino acids are summarized in Table 2. Compared with the reference CPV9 strain, both CPV9 sequences from Dog 1 and Dog 2 contained a 27 bp insertion at nucleotide position 1113 of the reference strain in the E1 gene, resulting in nine additional amino acids (a.a.) in the E1 protein. Interestingly, this additional short nine a.a. insertion shared 89% (24/27) of its sequence identity with the E1 of CPV12 (Accession no. JQ754321.1).

Compared with the E2 gene of the CPV9 derived from the benign lesion of Dog 2, which shared the same length nucleotide sequence and 99.9% of its identity with the reference strain, the CPV9 derived from the malignant SCC lesion of Dog 1 consisted of a 328 bp deletion spanning the nucleotide positions 4037–4364 of the reference strain. This deletion might contribute to a truncated E2 protein with a 67 a.a. deletion at the terminus of the E2 protein, including the removal of the original stop codon, resulting in an additional 11 a.a. at the C-terminus of the E2 protein. Furthermore, the E8^E2 mRNA transcript of Dog 1 was also affected by this deletion.

Sequence alignment of each ORF of all three CPV9 strains revealed identical lengths of E4, E6, E7, L1, and L2 genes with high nucleotide sequence identities among each other, ranging from 99.4–100%. While most of the mutations were synonymous mutations, three non-synonymous mutations, T350S in L1 and I229T in L2 of the CPV9 strain derived from Dog 2, and D476G in L2 of the CPV9 strain derived from Dog 1 were observed.

### 3.4. Detection of E6 and E7 mRNA by conventional RT-PCR

**Figure 4:**
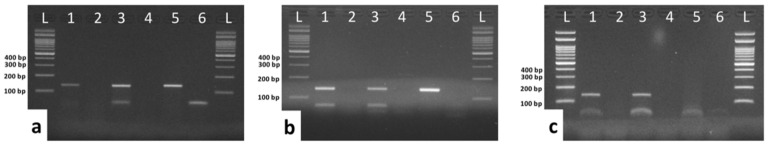
The detection of E6 and E7 mRNA expression in FFPE tissue from Dog 1 and Dog 2 via RT-PCR. The expected sizes of each primer pair targeting E6, E7, and RPS5 were 130 bp,137 bp, and 141 bp, respectively. Viral genomic DNA was used as a positive control for verifying the PCR conditions of E6 and E7. The reactions performed without templates were used as negative controls for the PCR reactions. To confirm that there was no viral genomic DNA contaminating the mRNA samples and subsequently affecting the results, after complete DNA digestion the mRNA samples from Dog 1 and Dog 2 were also used as negative controls. (**a**) Detection of E6 mRNA using primer-specific RT-PCR with mRNA-derived cDNA transcripts. (**b**) Detection of E7 mRNA using primer-specific RT-PCR with mRNA-derived cDNA transcripts. (**c**) The detection of the internal control, RPS5, mRNA using primer-specific RT-PCR with mRNA-derived cDNA transcripts. Lane 1: mRNA expression of Dog 1; Lane 2: negative control for excluding DNA contamination of the Dog 1 sample; Lane 3: mRNA expression of Dog 2; Lane 4: negative control for excluding DNA contamination of the Dog 2 sample; Lane 5: positive controls for E6 or E7; Lane 6: negative control for PCR reactions; L: DNA ladder.

Serving as oncogenic proteins, the mRNA expression of E6 and E7 in the tissues of both Dog 1 and Dog 2 was evaluated. As shown in Figure 4, the mRNA of E6 (Figure 4a), E7 (Figure 4b), and the internal control, RPS5 (Figure 4c), were successfully detected in both Dog 1 and Dog 2 via primer-specific RT-PCR. The expected sizes of the amplicons for E6, E7, and RPS5 were 130 bp, 137 bp, and 141 bp, respectively. The viral genomic DNA was used as a positive control for verifying the PCR conditions. No residual contamination of viral genomic DNA was detected in the mRNA samples after complete DNA digestion (before reverse transcription; Figure 4). Semi-quantitative data revealed no differences in the expression levels of E6 and E7 between Dog 1 and Dog 2.

### 3.5. Comparison of mRNA Expression Levels of E6 and E7 Using Quantitative PCR (qPCR)

To quantify and compare the expression levels of E6 and E7 between the two CPV9 strains from recurrent SCCs of Dog 1 and viral plaque of the Dog 2, qPCRs for detecting E6, E7, and the internal control, RPS5, were set up. Similar to the results from the conventional RT-PCR, no difference in the expression levels of E6 and E7 between Dog 1 and Dog 2 was noted, as shown in Supplementary Table S3.

## 4. Discussion

The association of viral oncoproteins with malignant transformation has been reported in HPVs. Several viral proteins, especially the E6 and E7 proteins, are suggested to contribute to the oncogenesis of HPVs in malignant carcinomas [36,37,38]. In the present study, we compared the full genomic sequences of a pair of CPV9 strains from two dogs diagnosed with SCC and benign viral plaque; this identified a unique 328 bp deletion at the C-terminal end of the E2 gene and spacer sequence, deduced to be truncated proteins of E2 and E2^E8 of the CPV9 from the dog with recurrent cutaneous SCC. To address the possible oncogenesis of the deleted-CPV9 strain, the mRNA expression levels of two major oncoproteins, E6 and E7, were evaluated by RT-PCR and RT-qPCR. However, there was no difference in the expression levels of E6 and E7 between the tissues infected with different strains of CPV9. These results suggest that the E2 or E2^E8 protein might play a role in the oncogenesis of CPV9. However, the effect of E2 and E8^E2 deleted CPV9 on the oncogenesis of benign and malignant lesions might be different from that of HPVs, and the actual mechanism should be investigated further.

In the present study, based on the sequence of the L1 gene, which encodes the major capsid L1 protein, both of our CPV9 strains share more than 99% identity with that of the reference CPV9 strain and were classified in the same genotype as CPV9 [39]. However, some nucleotide diversity and consequent amino acid substitutions were observed in E1, E2, E2^E8, E4, L1, and L2 of our CPV strains; an identical short insertion adding an additional nine a.a. on E1 compared to the published reference strain was also noted in both of our CPV9 strains. Interestingly, the nucleotide insertion shared 89% (24/27) and 81% (22/27) nucleotide identity with the reference strains of CPV12 (JQ754321.1) and CPV20 (KT901797.1), respectively [40,41,42]. Since the CPV9 strain from the SCC of Dog 1 and from the benign lesions of Dog 2 were sequenced from different labs located in Taiwan and Japan, respectively, the insertion of nine a.a. in the E1 protein different from the United States reference strain of CPV9 has been concurrently confirmed. We speculated that the E1 insertion in CPV9 identified in this study might be due to the occasional inter-species and intra-species recombination events of PVs [43,44]. The recombination of the E1 gene among HPV157, HPV158, and HPV209 has been demonstrated in HPVs [45]. Further investigations should study the potential evidence of inter-species recombination of CPVs.

Several viral proteins have been reported to be associated with carcinomas in HPVs [1,15]. Epithelial papillomavirus infections, even those with the same genotype, might lead to different clinical outcomes. For oncogenesis of HPVs, E2 and/or E2^E8 can repress the promoters of the oncoproteins, E6 and E7, and thus is key to limiting the expression levels of E6 and E7 in oncogenic HPVs [46]. It has also been demonstrated that the deletion of E2 or the abrogation of the DNA-binding site on E2 will contribute to the increased expression of the E6 and E7 oncoproteins, and the E2^E8 deletion may increase virus replication and transformation in the long term for certain HPVs and BPV1 [36,37,38]. Similar to the HPVs, a large deletion was also identified at the C-terminus of the E2 open reading frame of the CPV9 derived from the SCC in Dog 1, but was not observed in the CPV9 from the viral plaque of Dog 2 [46,47].

Since the E2-deleted CPV9 was identified from the dog with SCCs (Dog 1), the association between the mutated E2 protein and oncogenesis was addressed by evaluating the expression levels of the well-known oncoproteins E6 and E7. Due to the lack of specific antibodies, the expression levels of E6 and E7 were alternatively estimated by detecting mRNA expression levels using real-time RT-PCR. However, the mRNA expression levels of E6 and E7 in the tissues of both SCC (Dog 1) and viral plaque (Dog 2) were not different. Therefore, the carcinogenesis of E2-deleted CPV9 remains unclear. The published information about E2, E6, and E7 was based on the study of HPVs and BPVs, and the protein functions of CPVs have rarely been discussed. Further proteomic functional studies are needed to verify the oncogenesis of CPVs.

## 5. Conclusions

Two different CPV9 strains were identified from benign or malignant skin lesions. Both CPV9 strains contained a 27 bp nucleotide in-frame insertion on the E1 gene. Only the CPV9 stain from the malignant skin lesion had a large deletion at the C-terminus of the E2 gene, leading a deduced truncated E2 and E2^E8 proteins. The E2 and E2^E8 can repress the promoters of the oncoproteins, E6 and E7, therefore, the mRNA expression levels of E6 and E7 were also estimated by RT-PCR and qRT-PCR. However, there was no difference in the mRNA expression levels between two CPV9 strains.

## Figures and Tables

**Table 1:** The positions of each open reading frames (ORF) and the corresponding lengths of each gene and protein.

ORF	CPV9 Strain	Location	Gene Length	Protein Length
E6	Dog 1	263–718	456 bp	151 a.a.
	Dog 2			
	JF800656.1			
E7	Dog 1	678–980	303 bp	100 a.a.
	Dog 2			
	JF800656.1			
E1	Dog 1	970–2862	1893 bp	630 a.a.
	Dog 2	970–2862	1893 bp	630 a.a.
	JF800656.1	970–2835	1866 bp	621 a.a.
E2	Dog 1	2804–4099	1296 bp	431 a.a.
	Dog 2	2804–4267	1464 bp	487 a.a.
	JF800656.1	2777–4240	1464 bp	487 a.a.
E8^E2	Dog 1	1352–1389^3409–4099	729 bp	242 a.a.
	Dog 2	1352–1389^3409–4267	897 bp	298 a.a.
	JF800656.1	1325–1362^3382–4240	897 bp	298 a.a.
E4	Dog 1	3375–4028	654 bp	217 a.a.
	Dog 2	3375–4028		
	JF800656.1	3348–4001		
L2	Dog 1	4180–5697	1518 bp	505 a.a.
	Dog 2	4508–6025		
	JF800656.1	4481–5998		
L1	Dog 1	5750–7255	1506 bp	502 a.a.
	Dog 2	6078–7583		
	JF800656.1	6051–7556		

a.a.: amino acid.

**Table 2:** The summary of point mutations, insertion, and deletion of DNA sequence and its corresponding deduced amino acids. The substitution of amino acid was labeled with *.

Point Mutations						
Position	Reference Strain (JF800656.1)		Dog 1 (MT265225)		Dog 2 (MT265226)	
	Nucleotide	Amino Acid	Nucleotide	Amino Acid	Nucleotide	Amino Acid
3806	C	D	T	D	T	D
4579	T	I	T	I	C	I
4927	C	S	C	S	T	S
4987	C	S	C	S	T	S
5011	A	V	G	V	A	V
5080	T	T	A	T	T	T
5166	T	I	T	I	C	T*
5228	C	R	A	R	A	R
5233	T	R	G	R	T	R
5281	C	P	C	P	T	P
5910	A	D	G	G*	A	D
6125	C	D	C	D	T	D
6128	T	S	T	S	A	S
6722	A	V	T	V	A	V
6779	G	G	A	G	G	G
7098	A	T	A	T	T	S*
7343	G	P	A	P	G	P
7542	C	R	C	R	A	R
Insertion						
1113	Reference Strain		G			
	Dog 1		TAGCTTGGAGGGATCAA ATAGTCAGGGG			
	Dog 2		CAGCTTGGAGGGATCAAATA GTCAGGGG			
Deletion						
4037–4364	Dog 1		328 bp deletion			
	Dog 2		No deletion			

## Supplementary Materials

The following are available online at https://www.mdpi.com/1999-4915/12/7/736/s1: Supplementary Table S1: The primers used in full genome sequencing from fresh tissue of Dog 1; Supplementary Table S2: The primers used in full genome sequencing from formalin-fixed paraffin-embedded (FFPE) tissue of Dog 2; Supplementary Table 3: The evaluation of mRNA expression levels of E6 and E7 using quantitative PCR (qPCR).
