# Supplementary material for: Whole Genomic Analysis and Comparison of Two Canine Papillomavirus Type 9 Strains in Malignant and Benign Skin Lesions
Source: Viruses. 2020 Jul 8;12(7):736. doi: 10.3390/v12070736 (PMC7412457; doi:10.3390/v12070736)
Supplement: Supplementary file 1 [file viruses-12-00736-s001.pdf]

viruses

Whole Genomic Analysis and Comparison of Two Canine Papillomavirus Type 9 Strains in Malignant and Benign Skin Lesions

Chia-Yu Chang[1,2], Nanako Yamashita-Kawanishi[3], Sonoka Tomizawa[3], I-Li Liu[1,4], Wei-Tao Chen[1], Yen-Chen Chang[1,2], Wei-Hsiang Huang[1,2]; Pei-Shiue Tsai[1], Kinji Shirota[5], James K Chambers[6], Kazuyuki Uchida[6], Takeshi Haga[3*] and Hui-Wen Chang[1,2*]

Supplementary Table 1. The primers used in full genome sequencing of the CPV 9 in the fresh tissue of Dog 1.

Primer pair	Region	Primer sequence	Amplicon
1.	7277–1053	Forward: 5′-TTTGCAGTTGCGTGCTCTGT-3′<br>Reverse: 5′-ACTACACTCCGCTTCCCTCA-3′	1349 bp
2.	900–2916	Forward: 5′- CTAGAAAGCCTTCTGTGCCGA-3′<br>Reverse: 5′- TTGCGCATCAGGTTCCAAT-3′	2017 bp
3.	2638–4729	Forward: 5′- TGGCGCTACCTATACAGTAGGA-3′<br>Reverse: 5′- TAGAGTGTTGCGACCAGCTT-3′	2092 bp
4.	4649–6016	Forward: 5′- CAGTGACAGTGCATGCGGTA-3′<br>Reverse: 5′- AGGACGCTCGGAAAAAGCAA-3′	1368 bp
5.	5421–7378	Forward: 5′- CTTCGAAAACCTGCGGTTGG-3′<br>Reverse: 5′- TGCGGTTTACGGTGGAATGT-3′	1958 bp

Supplementary Table 2. The primers used in full genome sequencing of CPV9 in the formalin-fixed paraffin-embedded (FFPE) tissue of Dog 2.

Primer pair.	Region	Primer sequence	Amplicon
1.	1–429	Forward: 5′-CTGCATTGAGCCAGACCGTT-3′<br>Reverse 5′- GCGTGCGGACGGTTATTTT-3′	430 bp
2.	327–881	Forward: 5′-AAGTACCGTGCGTGTTCTG-3′<br>Reverse: 5′- TGTGCAGAGGACCACAATCC-3′	555 bp
3.	788–1258	Forward: 5′-GGAGTCGCCTCCCAATCAAA-3′<br>Reverse: 5′- GCCTAGGGCTTAGGTAGGCA-3′	471 bp
4.	1034–1542	Forward: 5′-TGAGGGAAGCGGAGTGTAGT-3′<br>Reverse: 5′- GAACAGTGCATACTGCGTCG-3′	509 bp
5.	1379–1817	Forward: 5′-ACAACGACCAGGTAGATGCC-3′<br>Reverse: 5′-GCCCCCGCAACATTCAATAC-3′	439 bp
6.	1629–2170	Forward: 5′- ATTCGGGGTGCACCATAACA-3′<br>Reverse: 5′- TTGTAGCCATCATTGCCCGT-3′	542 bp
7.	1849–2407	Forward: 5′-AGTGTTGCAGCAGCCCTATT-3′<br>Reverse: 5′-TGACCTTGCCCCCGAAAAAT-3′	559 bp
8.	2094–2705	Forward: 5′-CACAAACTCACAGGCGAAGC-3′<br>Reverse: 5′-CCCGCCTCATTAAAGGGGAA-3′	612 bp
9.	2525–3132	Forward: 5′-ATGCACTGGACGGAAACACA-3′<br>Reverse: 5′-AAACAGTTAGAGGGCTCGGC-3′	608 bp
10.	2950–3367	Forward: 5′-AGGAATTATCCGGCTTGGGC-3′<br>Reverse: 5′-CATCACATCCCACTGACCCC-3′	418 bp
11.	3258–3740	Forward: 5′-ATGTGAGTGGCGAAGGACTC-3′<br>Reverse: 5′-CTGGGACCTGGGAAACCAAA-3′	483 bp
12.	3602–4109	Forward: 5′-GCACAGGAGCAGATACACCC-3′<br>Reverse: 5′-TATTGCAGAAAAGGCCCCGA-3′	508 bp
13.	3937–4483	Forward: 5′-AGGTTCAGAAGGACGCTTTC-3′<br>Reverse: 5′-GGGTATCCTCTGCGCG-3′	547 bp
14.	4341–4752	Forward: 5′-CATGTTTCCCGTTGCACCTC-3′<br>Reverse: 5′- ACTGTCACCCCTGTACCTGT-3′	412 bp
15.	4520–5054	Forward: 5′-CGTAGACTTCCCAGAGCGTC-3′<br>Reverse: 5′-AGTGTTGCGACCAGCTTACA-3′	535 bp
16.	4799–5304	Forward: 5′-GTTGGCGCCACTGATGTTTT-3′<br>Reverse: 5′-TCCACAACTGCACTGAGGTC-3′	506 bp
17.	5035–5490	Forward: 5′-TGTAAGCTGGTCGCAACACT-3′<br>Reverse: 5′-ATACCATGTCTGCCAACCCG-3′	456 bp
18.	5309–5790	Forward: 5′-GGACCACGTGTTGTGTTTGG-3′<br>Reverse: 5′-CTGTTGCGCCCTCTAGGAAA-3′	482 bp
19.	5450–6094	Forward: 5′-CTTTCGGAGTCTGGCCATGT-3′<br>Reverse: 5′-GGTACCCACACGGACATCTG-3′	645 bp
20.	5767–6345	Forward: 5′-GGAATTTCCTAGAGGGCGCA-3′<br>Reverse: 5′-TAGGACGCTCGGAAAAAGCA-3′	579 bp
21.	6196–6682	Forward: 5′-ACAGGCTTCTAACAGTGGGC-3′<br>Reverse: 5′-TCAAGCATGTCACCGTCCTG-3′	487 bp
22.	6324–6903	Forward: 5′-TTTGCTTTTTCCGAGCGTCC-3′<br>Reverse: 5′-ATAGGGAGGAGGGTACAGGC-3′	580 bp
23.	6823–7480	Forward: 5′-TTGCAAGACGGGAACAGGAA-3′<br>Reverse: 5′-CGTCCAAGAGGGAACTGGTC-3′	658 bp
24.	7367–7846	Forward: 5′-TCCGGATAAGGCTCCTCCAA-3′<br>Reverse: 5′-AAGTTGATGCACACCTGGCT-3′	480 bp
25.	7636–252	Forward: 5′-CTCCTGTGTAGTCAGCAGGC-3′<br>Reverse: 5′-GCAACGGTTTCGTGGCAATA-3′	517 bp

Supplementary Table 3. The evaluation of mRNA expression levels of E6 and E7 using quantitative PCR (qPCR). The dog ribosomal protein S5 (RPS5) was included as the internal control for the estimation of mRNA expression levels of E6 and E7.

	**mRNA expression of E6**		mRNA expression of E7		mRNA expression of RPS5	
	**Cycle number**	**Melting temperature**	Cycle number	Melting temperature	Cycle number	Melting temperature
Dog 1	25.95	82.0 ◦C	26.41	83.5 ◦C	27.00	85.5 ◦C
Dog 2	26.07	82.0 ◦C	26.35	83.5 ◦C	27.01	85.5 ◦C
